# A Nanoscale Study of Carbon and Nitrogen Fluxes in Mats of Purple Sulfur Bacteria: Implications for Carbon Cycling at the Surface of Coastal Sediments

**DOI:** 10.3389/fmicb.2017.01995

**Published:** 2017-10-24

**Authors:** Cédric Hubas, Dominique Boeuf, Bruno Jesus, Najet Thiney, Yann Bozec, Christian Jeanthon

**Affiliations:** ^1^Muséum National d’Histoire Naturelle, UMR BOREA, MNHN-CNRS-UCN-UPMC-IRD-UA, Station de Biologie Marine de Concarneau, Concarneau, France; ^2^CNRS, Station Biologique de Roscoff, Adaptation et Diversité en Milieu Marin, Roscoff, France; ^3^Sorbonne Universités, UPMC Univ Paris 06, UMR 7144, Station Biologique de Roscoff, Adaptation et Diversité en Milieu Marin, Roscoff, France; ^4^EA2160, Laboratoire Mer Molécules Santé, Université de Nantes, Nantes, France; ^5^BioISI – Biosystems & Integrative Sciences Institute, Campo Grande University of Lisbon, Faculty of Sciences, Lisbon, Portugal; ^6^Muséum National d’Histoire Naturelle, UMR BOREA, MNHN-CNRS-UCN-UPMC-IRD-UA, Bâtiment Arthropodes, Paris, France

**Keywords:** microbial mats, *Chromatiaceae*, Thiohalocapsa, stable isotope probing (SIP), NanoSIMS, compound specific isotope analysis (CSIA), fatty acid methyl ester (FAME)

## Abstract

Mass blooms of purple sulfur bacteria growing seasonally on green stranded macroalgae have a major impact on the microbial composition and functionality of intertidal mats. To explore the active anoxygenic phototrophic community in purple bacterial mats from the Roscoff Aber Bay (Brittany, France), we conducted a combined approach including molecular and high-resolution secondary ion mass spectrometry (NanoSIMS) analyses. To investigate the dynamics of carbon and nitrogen assimilation activities, NanoSIMS was coupled with a stable isotope probing (SIP) experiment and a compound specific isotope analysis (CSIA) of fatty acid methyl ester (FAME). Sediment samples were incubated with ^13^C- and/or ^15^N-labeled acetate, pyruvate, bicarbonate and ammonium. NanoSIMS analysis of ^13^C - and ^15^N -incubated samples showed elevated incorporations of ^13^C - and ^15^N in the light and of ^13^C -acetate in the dark into dense populations of spherical cells that unambiguously dominated the mats. These results confirmed CSIA data that ranked vaccenic acid, an unambiguous marker of purple sulfur bacteria, as the most strongly enriched in the light after ^13^C -acetate amendment and indicated that acetate uptake, the most active in the mat, was not light-dependent. Analysis of DNA- and cDNA-derived *pufM* gene sequences revealed that *Thiohalocapsa*-related clones dominated both libraries and were the most photosynthetically active members of the mat samples. This study provides novel insights into the contribution of purple sulfur bacteria to the carbon cycle during their seasonal developments at the sediment surface in the intertidal zone.

## Introduction

Microbial mats are complex small-scale self-sustaining benthic ecosystems (Stal, 1985) often built by cyanobacteria whose primary production enrich the sediment with organic matter that becomes available to different functional groups of microorganisms. In such ecosystems, the surface sediment layer (a few millimeters thick) is a transition zone between oxic and anoxic conditions that favor the development of stratified and cohesive layers of diverse assemblages of oxygenic and anoxygenic phototrophic microorganisms. Mass blooms of anoxygenic phototrophic bacteria, forming large colored mats, can occur at the sediment surface in the intertidal zone (Imhoff, 2001). On the Orkney islands (Wieland et al., 2003) and Roscoff Aber Bay (Hubas et al., 2013), blooms dominated by purple sulfur bacteria typically occur on sandy sediments locally enriched in organic matter derived from the decomposition of macroalgal deposits. In their habitats, such as enclosed lagoons, estuaries, and salt marshes, purple sulfur bacteria have to cope with occasionally occurring drastic changes of salinity, temperature, and light conditions (Caumette, 1993; Caumette et al., 1994; Imhoff, 2014). This situation occurs in Roscoff Aber Bay, a temperate intertidal bay, where muddy-sandy sediments are extensively covered by stranded *Enteromorpha* sp. and mats of purple sulfur bacteria during warm summer months (Hubas et al., 2006b, 2011). In a preliminary study (Hubas et al., 2013), we reported that these mats were dominated by *Chromatiaceae*. These purple sulfur gammaproteobacteria are all capable of photolithoautotrophic growth under anoxic conditions with sulfide and elemental sulfur as electron donors and are potentially mixotrophic and photoassimilate simple organic compounds, of which acetate and pyruvate are the most widely used (Imhoff, 2006). We previously assessed the biochemical composition (fatty acids, photosynthetic pigments) of the microbial mats and their influence on ecosystem functions (sediment cohesiveness, CO_2_ fixation) at low tide in Roscoff Aber Bay and demonstrated that the proliferation of these purple sulfur bacteria have a major impact on the diversity and functionality of intertidal mats (Hubas et al., 2013). Particularly, their massive growth resulted in a dramatic increase of both gross CO_2_ fixation as well as total mat respiration in comparison to diatom-dominated mats. However, despite the increase of total CO_2_ fixation, purple sulfur bacterial mats presented a net CO_2_ degassing whereas diatom-dominated mats represented a net CO_2_ sink. We suggested that photosynthetic efficiency was probably hampered in these mats and that macroalgal-derived organic matter favored the photoheterotrophic lifestyle of purple sulfur bacteria.

Nanoscale secondary ion mass spectrometry (NanoSIMS) is a powerful technique capable of imaging elemental distributions and measuring metabolic activities of a single-cell level in mixed populations, including within biofilms. Combining NanoSIMS with stable isotope enriched incubations has been used recently to study the cycling and flux of carbon, sulfur and nitrogen in phototrophic microbial mats from several environments (Fike et al., 2008; Burow et al., 2012; Woebken et al., 2012, 2015; Lee et al., 2014). To better understand the structure and the development of phototrophic purple sulfur bacteria assemblages at the surface of intertidal sediment and their influence on important ecosystem functions and services, we sampled undisturbed cores of these mats and performed a stable isotope probing (SIP) experiment. In this study, our aims were (1) to identify the dominant and active members of the mats, (2) to elucidate preferred carbon sources taken up by the mat members and (3) to estimate organic and inorganic carbon and nitrogen uptake rates at the sub-micrometric scale by combining compound specific isotope analysis (CSIA) of fatty acid methyl ester (FAME) and NanoSIMS analyses.

## Materials and Methods

### Sampling Site

Sediment cores and microbial mats were collected at mid day in August 2010 at low tide in the Roscoff Aber Bay (Brittany, France; 48°42′59.836 N, 3°59′51.611 W). The bay is about 1 km long and 2 km wide, entirely situated above mid-tide level, and its annual benthic metabolism has been extensively studied in the past (Hubas and Davoult, 2006; Hubas et al., 2006b, 2007). Particularly sheltered, the bay is dominated by sandy-muddy sediments, with local freshwater inputs from the river located at the landward end of the bay and with freshwater seepages. The bay is affected by the seasonal proliferation of green macroalgae that affect both the trophic ecology (Riera and Hubas, 2003) and C fluxes of the ecosystem (Hubas and Davoult, 2006). The selected mats were established at the surface of fine-sand intertidal sediments (mean particle size: 215 ± 43 μm, Hubas et al., 2006b). Oxic layers were absent or very thin (<1 mm thick) below these mats.

### Incubation Experiments with Stable Isotopes

**FIGURE 1:**
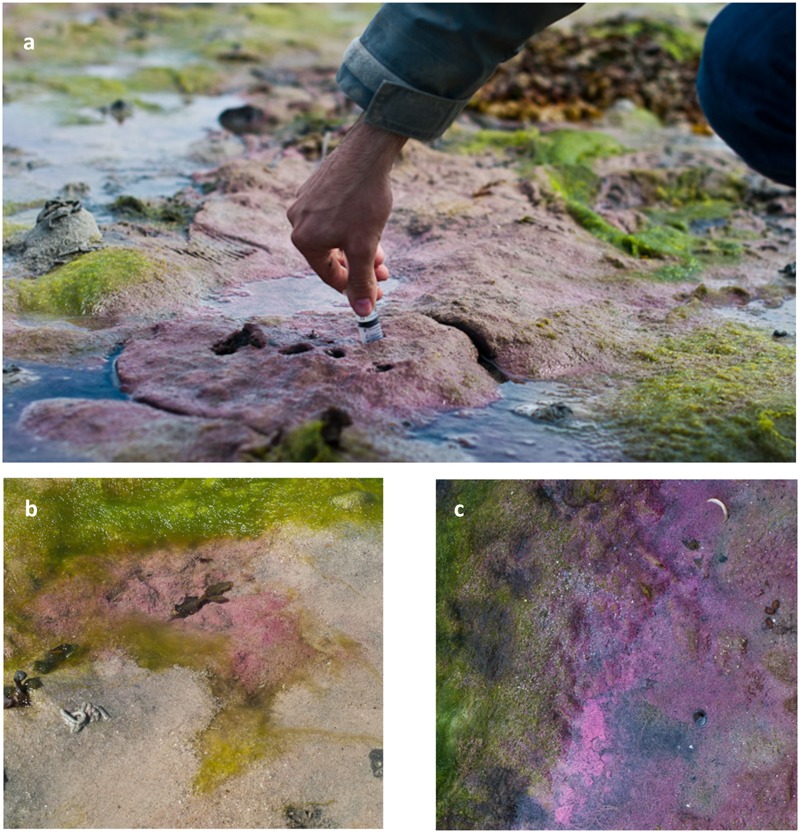
General aspect of various types of purple sulfur bacterial mats of the Roscoff Bay. Emersed **(a,b)** or immerged **(c)** biofilms. Adapted from Hubas et al. (2013).

Core incubations were first carried out to follow substrate incorporation in microbial fatty acids over time using specific isotope analysis of fatty acid methyl ester (CSIA-FAME). Undisturbed microbial mats were isolated at mid day from surrounding sediment using sterile polycarbonate cut-off syringes (10 mm diameter) pushed down to about 3 cm depth (**Figure 1**). Three cores used to determine baseline conditions (control) were immediately removed from the sediment; the first two millimeters were sampled and immediately frozen in liquid nitrogen. The remaining cores were used for 3 different triplicate incubations designated AceL, PyrL, CO3L. Incubation designations referred to the labeled substrate added and to the light condition: “Ace” for [1-^13^C] sodium acetate-1- (99 atom %), “Pyr” for [2-^13^C] sodium pyruvate (99 atom %) and “CO3” for ^13^C sodium bicarbonate (99 atom %), “L” for incubations at ambient light. A constant volume (1.9 ml) of the ^13^C labeled substrate solutions (final concentration of 500 μM, prepared with seawater freshly collected from the study site) was gently added on top of the mat cores and incubated *in situ* at ambient light (>1000 μmol photons m^-2^ s^-1^) and temperature (22–23°C) for 2 h. At the end of the incubation period, cores were removed from the sediment, the first two millimeters sampled and immediately frozen in liquid nitrogen.

Core incubations were also carried out to examine the assimilation of labeled substrates at single cell level by NanoSIMS analysis. Several underwater purple sulfur bacterial mats were sampled as described above, homogenized and brought back to laboratory shortly after collection under controlled conditions. Briefly, the homogenized microbial mats were used for 7 different triplicate incubations designated control, AceL, AceD, PyrL, PyrD, CO3L, and CO3D. Incubation designations were as above and “D” referred to incubations in the dark. The ^13^C labeled substrate solutions were prepared as above and incubations were performed at ambient light (>1000 μmol photons m-2 s-1) or in the dark at controlled temperature (19°C) for 5 h. Cores samples were incubated with ^13^C-labeled bicarbonate, ^13^C-labeled acetate and ^13^C -labeled pyruvate In addition, to estimate nitrogen uptake, ^15^N-labeled ammonium chloride (98 atom %; final concentration of 100 μM) was added to all incubations. The final atomic ratios (bicarbonate: 28.1, acetate: 33.5, pyruvate: 86.9% atom percent ^13^C, ammonium: 74.5% atom percent ^15^N) were calculated taking into account the natural concentrations of the corresponding substrate previously measured in discrete seawater samples collected before mat collection (bicarbonate, ammonium) or concentration ranges (acetate, pyruvate) used in similar studies (Morono et al., 2011; Tourna et al., 2011). After incubation, purple sulfur bacterial mats were fixed with 2% paraformaldehyde for 1 h, filtered on gold-coated polycarbonate (GTTP type; pore size 0.22 μm, diameter 25 mm, Millipore), and washed with filter-sterilized seawater to remove labeled substrates in excess. The filters were further stored at -20°C until processing.

#### Fatty Acid Composition

Fatty acids (FAs) were extracted following the method of Bligh and Dyer (Bligh and Dyer, 1959) slightly modified by Meziane et al. (2006). Total lipids were extracted by sonication (35 kHz, 20 min) using a chloroform/methanol/water cocktail (2:1:1, v:v:v), concentrated under a constant N_2_ flow, and the residue hydrolyzed (90 min, 90°C) with NaOH:MeOH (1:2, v:v). An internal standard (tricosanoic acid: 23:0, 10 μg) was added to each sample before extraction. Hydrolysis (i.e., saponification) aimed to release individual FAs from more complex molecules, such as triglycerides or phospholipids, by breaking the ester bonds. Individual FAs were then derivatized into fatty acids methyl esters (FAMEs) by the addition of one methyl (CH3) group (i.e., methylation procedure) so that they can be separated and quantified by gas chromatography. Methylation was performed by incubating individual FAs with boron-trifluoride methanol (BF3-MeOH) at 90°C for 10 min. Sample were then dried under N_2_ flux and transferred to hexane for injection in a gas chromatograph (GC, Varian CP-3800 equipped with flame ionization detector - FID). Most FAMEs were identified by comparing their retention times with those of known standards (Supelco^TM^ 37, PUFA-1 Marine Source, and Bacterial Mix; Supelco Inc., Bellefonte, PA, United States). Unidentified FAMEs were further identified by GC coupled to a mass spectrometer (GC-MS, Varian GC450-220MS). For both devices, FAMEs separation was performed using a Supelco OMEGAWAX 320 column (30 m × 0.32 mm i.d., 0.25 μm film thickness) with H_2_ as carrier gas. After injection of 1 μl of sample at 60°C, the temperature was raised to 150°C at 40°C min-1, then to 240°C (held 14 min) at 3°C min-1. FAMEs were systematically corrected for the added methyl group and corresponding individual FAs are designated in this study as CX:Y-nZ, where X is the number of carbons, Y the number of double bonds and Z the position of the ultimate double bond from the terminal methyl. Cyclopropane FAMEs were detected and noted 9-10diMe C16 and 9-10diMe C18 for Methyl cis-9,10-methylene hexa- and octa-decanoate, respectively.

### Compound Specific Isotope Analysis (CSIA) of Fatty Acid Methyl Ester (FAME)

After GC and GC-MS analyses, isotope ratio gas-chromatograph-mass spectrometry (GC-IRMS) was used to measure the carbon stable isotope ratios (expressed in ‰) of individual fatty acids. Measurements were performed at the UC Davis Stable Isotope Facility of the University of California (Davis, CA, United States). FAMEs dissolved in hexane are injected in splitless mode and separated on a Varian factorFOUR VF-5ms column (30 m × 0.25 mm ID, 0.25 micron film thickness). Once separated, FAMEs are quantitatively converted to CO_2_ in an oxidation reactor at 950°C. Following water removal through a nafion dryer, CO_2_ enters the IRMS. δ^13^values were corrected using working standards composed of several FAMEs calibrated against NIST standard reference materials. Stable carbon isotope ratios for individual fatty acids (FA) were calculated from FAME data by correcting for the one carbon atom in the methyl group that was added during the derivatization process. This correction was made according to Gladyshev et al. (2012) by taking into account the isotope ratio of the derivatized methanol (BF3-methanol, -37.9 ‰ in our study), and the fractional carbon contribution of the free fatty acid to the ester.

(1) $$\delta^{13}C_{FA}=\frac{\left(\delta^{13}C_{FAME}-(1-f)\cdot \delta^{13}C_{CH_{3}OH}\right)}{f}$$

where δ^13^C_FA_ is the isotopic composition of the free fatty acids, δ^13^C_FAME_ is the isotopic composition of the fatty acid methyl ester, *f* is the fractional carbon contribution of the free fatty acid to the ester and δ^13^C_CH3OH_ is the isotopic composition of the methanol derivatization reagent. For instance, in the case of a C16 FA, *f* would be equal to 16/17. The isotopic composition of the methanol was determined by the same GC-IRMS system.

### Molecular Identification of the Mat-Forming Purple Bacteria Using 16S rRNA and *pufM* Genes

Biomass from three microbial mats was collected close to where the cores were sampled using sterile plastic syringes and transferred in Eppendorf tubes. Samples were immediately frozen in liquid nitrogen and further stored at -80°C until analysis. For DNA extraction, cells were thawed and suspended in DNA lysis buffer (0.75 M sucrose, 50 mM Tris-HCl, pH 8) and processed using the procedure described by Marie et al. (2006). Total RNA was extracted using the RNeasy Mini kit (Qiagen) following the manufacturer’s instructions. Any trace of genomic DNA was removed using a Turbo DNA-free kit (Ambion). DNA removal in RNA samples was confirmed by control PCR amplifications without the reverse transcription step. No amplification was detected in these controls. ThermoScript RT-PCR system (Invitrogen) was used for the reverse transcription of mRNA from total RNA samples. The cDNA synthesis was performed at 50°C using random primers.

Small-subunit (16S) rRNA genes were amplified by polymerase chain reaction (PCR) using universal reverse primer 1492R and Bacteria-specific forward primer 8F (Weisburg et al., 1991). PCR amplification of *pufM* genes was performed using the primers 557F and 750R designed by Achenbach et al. (2001). Reaction mixture (25 μL) contained the following components: 5X buffer (10 μl), 2 mM MgCl2, 10 pmoles of each deoxyribonucleotide triphosphate (dATP, dCTP, dGTP, dTTP; Eurogentec), 10 pmoles of each oligonucleotide primer, 2.5 U of GoTaq Flexi DNA polymerase (Promega) and 50 to 100 ng of template DNA. Amplifications were carried out in a GeneAmp PCR system 9700 (Applied Biosystems, Foster City, CA, United States) with the following parameters: 95°C for 5 min, followed by 35 cycles of 95°C for 30 s, annealing at 55°C, respectively, and extension at 72°C for 60 s and 30 s for 16S rRNA and *pufM* gene amplification, respectively, with a final extension step at 72°C for 10 min. Amplicons were cloned directly or after gel extraction using the TOPO4-TA cloning kit (Invitrogen) according to the manufacturer’s instructions. Clones were sequenced using an ABI 3130 POP7 sequencer (Applied Biosystems) at the Biogenouest Sequencing-Genotyping Platform (Roscoff site).

The sequences were trimmed to remove any vector and primer sequences. Chimeras were removed using Uchime (v4.2.40; Edgar et al., 2011). Gene sequences were compared to sequences in public databases with BLASTn (Altschul et al., 1997). The 16S rRNA gene sequences were aligned the SILVA SSUref N99 references (v.128) using ARB aligner. The *pufM* DNA sequences were translated into amino acid sequences and aligned using the MAFFT E-INS-I algorithm. The resulting protein alignment was back-translated to nucleotide acid sequences using pal2nal (Suyama et al., 2006). Conservative values of 97% and 94% nucleic acid sequence similarity for 16S rRNA and *pufM* sequences, respectively, were chosen for clustering sequences into Operational Taxonomic Units (OTUs) using MOTHUR (Schloss et al., 2009). Representative sequences (defined as the sequence with the minimum distance to all other sequences in the OTU) were obtained using MOTHUR.

A *pufM* database and a consensus Bayesian tree were built as described in Boeuf et al. (2013). Representative sequences of each OTU (245 pb) and short *pufM* environmental reference sequences were aligned as above and added to the backbone tree using the ADD_BY_PARSIMONY algorithm implemented in ARB software (Ludwig et al., 2004). Likewise, representative ssu sequences of each OTU (927 pb) were added to the SILVA reference tree using ARB. Non-informative taxa were removed from both final tree.

### NanoSIMS Image Acquisition

Secondary ion images of ^12^C-, ^13^C-, ^12^C^14^N-, ^12^C^15^N-, and ^32^S- were recorded simultaneously for each individual cell using the Cameca NanoSIMS N50 at the Muséum National d’Histoire Naturelle (Paris, France). All measurements were performed using the same analytical conditions. Firstly, a 30 × 30 μm image field was chosen and the filters were pre-sputtered during 4 min with a 18.8 pA Cs- primary ion beam that was stepped over the sample in a 256 × 256 pixel raster with a counting time of 1 ms per pixel. Then image acquisition was made by rescanning 25–30 times a 25 × 25 μm image field sputtered with a 0.5 pA Cs- beam in a 256 × 256 pixel raster with a counting time of 1 ms per pixel.

### NanoSIMS Data Processing

NanoSIMS data were then processed using the proprietary WinImage Software (L’image, CAMECA SIMS Image Processing). Purple sulfur bacteria were detected based on their typical coccoïd shape from the ^12^C^14^N- images and each bacterial cell was defined as a region of interest (ROIs). ROIs data were exported as.csv files and processed using the R© statistical framework to calculate carbon and nitrogen stable isotope ratios as well as atom percent of ^13^C and ^15^N.

(2) $$r_{i}=\frac{\left(R_{\text{ini}}-R_{\text{measured}.i}\right)}{R_{\text{ini}}-R_{\text{final}.i}}$$

where *r*_i_ is a dimensionless ratio for incubation experiment i, R_ini_ is the initial isotopic ratio of the bacteria (based on the mean of natural isotopic composition of the control), *R*_measured.i_ is the measured isotopic ratio (NanoSIMS) of a given bacterial cell for incubation experiment i, *R*_final.i_ is the theoretical isotopic ratio of a given bacterial cell based on the isotopic composition of the substrate used during the incubation experiment. *R*_final.i_ was calculated by taking into account the isotopic ratio of the substrate at its final concentration in the sample. It is assumed that no isotopic fractionation would occur during substrate assimilation. All isotopic ratios (R) are in atom %. Substrate incorporation was calculated as follows:

(3) $$F_{i}=\frac{C_{i}\cdot V_{\text{bac}}\cdot r_{i}}{t}$$

where *F*_i_ is the quantity of substrate incorporated by a bacterial cell per hour (moles.cell^-1^. h^-1^), C_i_ is the final concentration of substrate i (in moles.L^-1^), Vbac (in L) is the mean cell volume (estimated to 2.4 μm^3^, mean diameter = 1.67 μm), *r*_i_ is the dimensionless ratio for incubation experiment i (see Equation 2), *t* is the incubation time in h. *F*_i_ was then converted to carbon and nitrogen weight units (fg C.cell^-1^. h^-1^ and fg N.cell^-1^. h^-1^) assuming a molar mass of 12 and 14 g.mol^-1^ for carbon and nitrogen, respectively.

### Statistical Analyses

All analyses and graphs were performed using the R© statistical framework. Normality was checked to perform one sample *t*-tests. When normality and/or homogeneity of variance assumption were not met, Van der Waerden tests were performed, which convert the ranks of a non-parametric Kruskal–Wallis rank sum test into quantiles of the standard normal distribution. Analysis of covariance (ANCOVA) was performed to compare the regression slopes between carbon and nitrogen isotopic ratios. This analysis was used to test the effect of a categorical factor (i.e., treatment) on a dependent variable (i.e., N isotopic ratio) while controlling for the effect of a continuous co-variable (i.e., C isotopic ratio).

### Nucleotide Accession Numbers

16S rRNA gene and transcript sequences obtained in this study are deposited under GenBank accession numbers MF320555 to MF320732. Sequences of *pufM* genes and transcripts are deposited under GenBank accession numbers KX352089 to KX352145 and KX358536 to KX358562, respectively.

## Results

### Fatty Acid Composition

**FIGURE 2:**
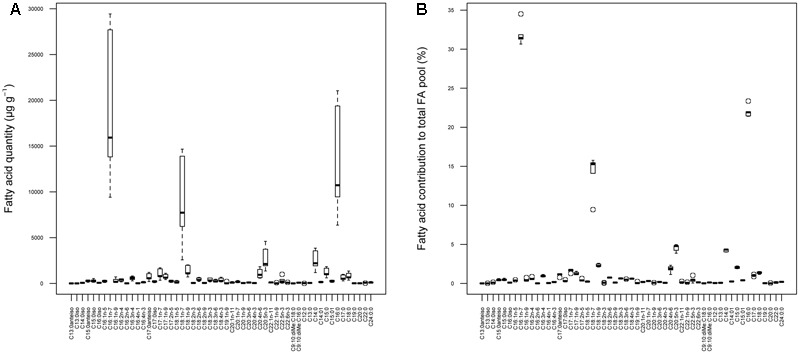
Fatty acid composition of the purple sulfur bacterial mats in terms of quantity **(A)** and percentages **(B).**

The mats were characterized by several bacterial markers such as branched FAs (i.e., iso and anteiso FAs), two cyclopropane FAs, as well as the vaccenic fatty acid (C18:1n-7). C16:1n-7, C18:1n-7, C16:0 and C14:0 prevailed in the mats and their concentrations varied greatly between treatments (**Figure 2A**). However, their proportions in the six treatments did not vary significantly from the control (one sample *t*-test, *p* > 0.05) (**Figure 2B**), indicating that treatments did not affect FA composition.

### Compound Specific Isotope Analysis (CSIA)

**FIGURE 3:**
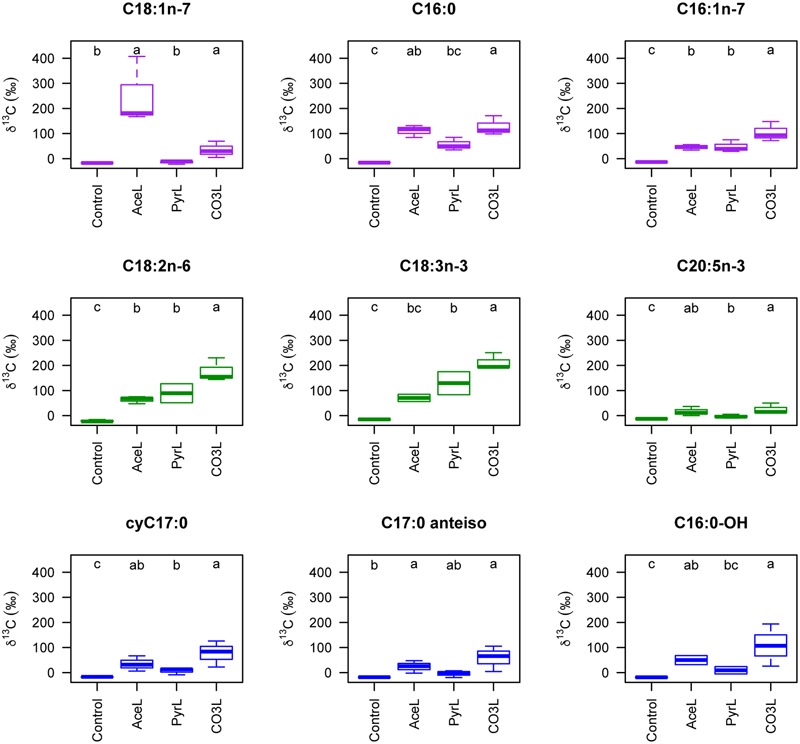
Isotopic ratio (δ^13^C notation) of the main fatty acids of purple sulfur bacterial mats. The **upper** (purple), middle (green) and **lower** (blue) panels corresponds to fatty acids which are considered representative of the purple sulfur bacteria, the macro- and microalgae, and bacteria. Note that the C18:1n-7 is considered as an unambiguous marker of purple sulfur bacteria. AceL, PyrL and CO3L correspond to the different treatments (respectively ^13^C-acetate, ^13^C-pyruvate and ^13^C-bicarbonate) at ambient light.

Sediment cores with dense purple sulfur bacterial mats were incubated at ambient light and temperature with ^13^C- and/or ^15^N-labeled acetate, pyruvate, bicarbonate and ammonium. Main cellular fatty acids of purple sulfur bacteria (Núñez-cardona, 2012) such as C18:1n-7, C16:1n-7 and C16:0 were detected by GC-IRMS and their isotopic compositions measured. According to Van der Waerden tests, fatty acids were systematically significantly enriched compared to the control (natural FA isotopic composition), when acetate and bicarbonate were used as growth substrates. Vaccenic acid (C18:1n-7), an unambiguous marker of purple sulfur bacteria was the most strongly enriched after ^13^C-acetate amendment. Although they were also significant, ^13^C enrichments with ^13^C-bicarbonate or ^13^C-pyruvate were much lower. In contrast, palmitic acid C16:0 and palmitoleic acid C16:1n-7 were more ^13^C enriched with bicarbonate than with acetate and pyruvate (**Figure 3**). Although algal fatty acids such as C18:2n-6, C18:3n-3 (macroalgae) and C20:5n-3 (microalgae) were significantly enriched by the selected substrates, they were more ^13^C enriched in the presence of bicarbonate. In addition to the bacterial markers identified by GC and GC-MS, several others were further identified by GC-IRMS such as another cyclopropane FA (cyC17:0), known to improve membrane fluidity of halophilic microorganims (Oren, 2002), and hydroxy fatty acids such as C16:0-OH (Wakeham et al., 2003). Both FAs and C17:0 anteiso were significantly more enriched when ^13^C-bicarbonate and ^13^C-acetate were provided as substrate than in the control and ^13^C-pyruvate amendment.

### Carbon and Ammonium Assimilation by Purple Bacterial Mats

**FIGURE 4:**
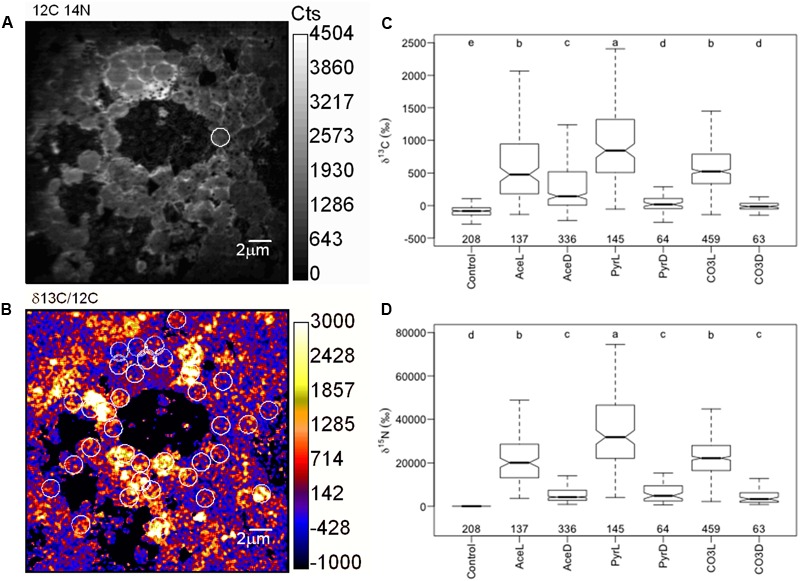
**(A,B)** Examples of NanoSIMS isotopic images of the purple sulfur bacteria mats. Purple sulfur bacteria were identified using the ^12^C^14^N-detector and each bacterial cell was defined as a region of interest (ROI: white circles). **(C,D)** Shows the carbon and nitrogen isotopic ratios (δ^13^C and δ^15^N notations) of the ROIs for the different treatments. Ace, Pyr and CO3 correspond to the different treatments (respectively ^13^C-acetate, ^13^C-pyruvate and ^13^C-bicarbonate), and “L” and “D” correspond to light and dark incubations, respectively. The total number of ROIs is given below each boxplot. Significant differences between treatments have been tested using a Van der Waerden test and the results are shown above each boxplot (treatments with the same letter are not significantly different).

**FIGURE 5:**
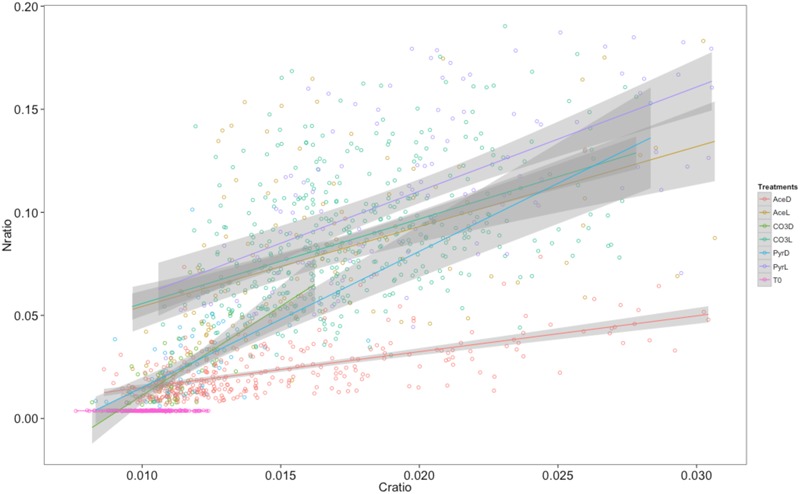
Relationships between nitrogen and carbon ratios of the different ROIs according to the different treatments. Each point represents a ROI (i.e., purple sulfur bacterial cell). Ace, Pyr and CO3 correspond to the different treatments (respectively ^13^C-acetate, ^13^C-pyruvate and ^13^C-bicarbonate), and “L” and “D” correspond to light and dark incubations, respectively. T0 corresponds to the control treatment. Regression lines were calculated and displayed on the graph for each treatment together with their confidence interval (gray surfaces).

**FIGURE 6:**
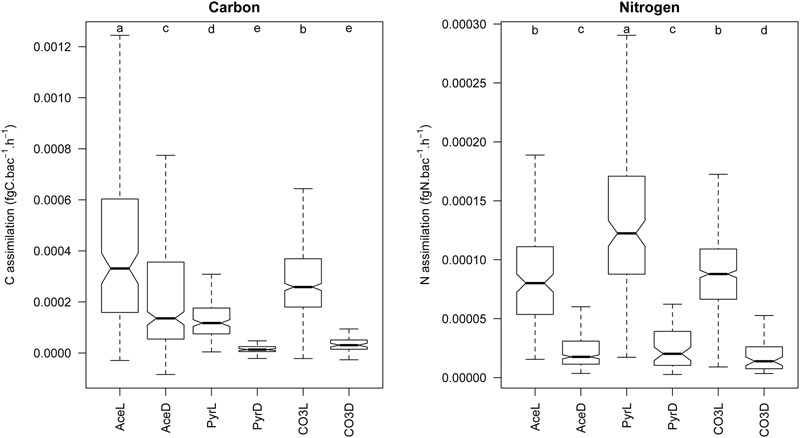
Assimilation rates of carbon and nitrogen at the single cell level according to the different treatments. Ace, Pyr and CO3 correspond to the different treatments (respectively ^13^C-acetate, ^13^C-pyruvate and ^13^C-bicarbonate), and “L” and “D” correspond to light and dark incubations, respectively. Significant differences between treatments have been tested using a Van der Waerden test and the results are shown above each boxplot (treatments with the same letter are not significantly different).

To evaluate how microbial populations actively assimilate organic/inorganic carbon and ammonium in the mats, we followed assimilation of ^13^C-acetate, ^13^C-pyruvate, ^13^C-bicarbonate and ^15^N-ammonium in individual cells in light and dark conditions and measure their uptakes in short-term experiments. NanoSIMS imaging revealed that the purple bacterial mats were formed by dense populations of coccoid cells aggregated in microcolonies. This dominant mat morphotype was detected based on its typical coccoid shape from the naturally abundant ^14^N (^12^C^14^N-) images (**Figure 4A**) and defined as ROI. A total of 1,204 bacterial cells were identified and encircled across all the treatments as shown in **Figure 4B**. For each ROI, ^15^N/^14^N (inferred from the ^12^C^15^N-/^12^C^14^N- ratio) and ^13^C/^12^C (^13^C-/^12^C-) ratios were calculated and expressed in delta value (‰, **Figures 4C,D**). Examination of the NanoSIMS images and delta values indicated that purple spherical bacterial cells were not equally enriched in ^15^N and ^13^C relative to the non-amended cells (control). Significant differences were observed between treatments (**Figures 4C,D**) and within treatments, especially under light. Cells displayed a great heterogeneity in terms of C and N uptake at a very small spatial scale (**Figure 4B**). They were substantially more enriched in both ^13^C and ^15^N under light that in the dark irrespective of the growth substrate. The highest delta values were obtained with pyruvate. A significant correlation between C and N ratios in each treatment (except control) as well as linear relationships (**Figure 5**) indicated that C and N uptakes were linked. All linear regressions, except AceD, were significantly similar (ANCOVA: *p* = 9.489e-12 with all treatments, *p* = 0.1037 when removing AceD, the control treatment was removed from all analyses). Assimilation rates were very variable and ranged from 0 to 0.002 fg C.bacterial cell^-1^. h^-1^ and from 0 to 0.0003 fg N.bacteria cell^-1^. h^-1^ (**Figure 6**). Uptake of ^13^C and ^15^N in the incubations were significantly higher in the light for the three carbon sources while no elevated ^13^C- and/or ^15^N-uptake was detected in dark incubations, except in ^13^C-acetate amendments, indicating that acetate incorporation, the most active in the dominant mat morphotype, was not light-dependent.

### Identification of the Main Phylotypes in the Purple Mats

The above described NanoSIMS analysis identified typical coccoid shaped purple bacteria highly active under light in incorporating acetate into biomass. To determine the bacterial community in the mats and identify which community members expressed the functional gene for anoxygenic photosynthesis, 16S rRNA and *pufM* clone libraries were constructed from DNA and cDNA recovered from two mat samples. Coverage estimates ranging from 66.7 to 98.4% (mean 82%) and rarefaction curves (Supplementary Figure S1) indicated that a large part of the sample diversity was detected in the libraries. In 16S rRNA clone libraries of two samples (178 clones), a substantial proportion of the sequences were related to the genera *Thiohalocapsa* (OTUs 8 and 22) and *Marichromatium* (OTUs 2 and related) within the *Chromatiaceae* family (Supplementary Figure S2). Both these genera, known to form dense purple layers in microbial mats, are morphologically distinct since *Thiohalocapsa* cells are spherical and cells of *Marichromatium* are straight to slightly curved rods (Caumette et al., 1991; Imhoff et al., 1998). The most abundant bacterial phylotype (OTU1, ∼48% of the total clones) in the libraries was, however, very closely related to a *Pseudomonas* strain isolated from a deep-sea sediment. The rest of the bacterial community was composed of minor OTUs that included sulfate-reducing bacteria (*Desulfobacteraceae*) and diverse members of the phylum *Bacteroidetes*.

**FIGURE 7:**
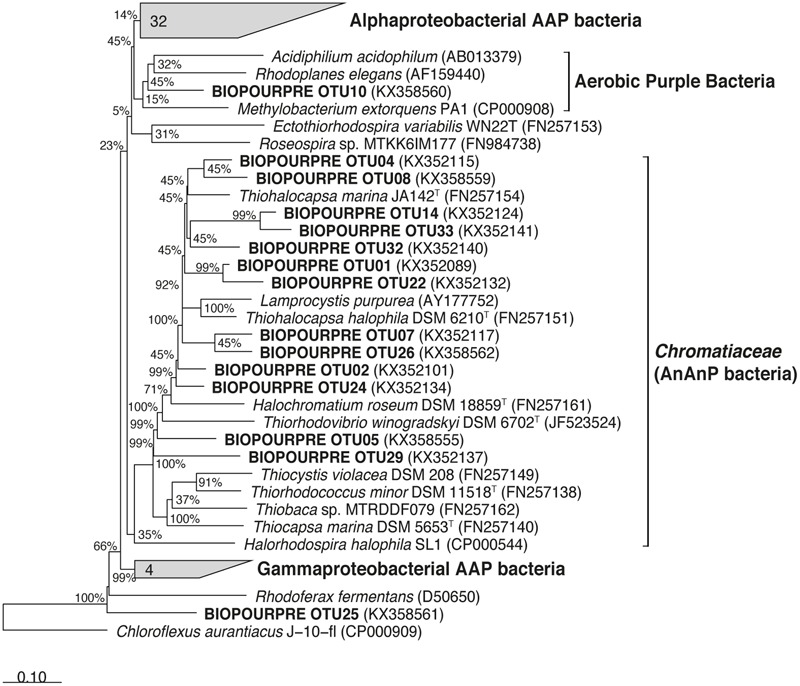
Phylogenetic tree showing the relationships between *pufM* gene sequences of anaerobic anoxygenic phototrophic bacteria retrieved from the Roscoff Aber Bay mats (in bold) and relatives retrieved in GenBank. The tree is based on a Bayesian tree to which sequences obtained in this study (189 bp) were added by ARB_PARSIMONY. The scale bar represents dissimilarity between nucleotide positions.

Most of the 203 total DNA- and cDNA-derived *pufM* sequences (95 and 108 clones, respectively) recovered from three mat samples were assigned to known genera of the *Chromatiaceae* (**Figure 7** and Supplementary Table S1). *Thiohalocapsa*-related *pufM* sequences were numerically dominant in the DNA libraries (>60% of the total sequences). Sequencing of the *pufM* transcripts revealed that the majority (>80%) of expressed *pufM* sequences recovered from cDNA of the samples also belonged to the *Thiohalocapsa* cluster, confirming the identity of the prevalent morphotype observed on NanoSIMS images.

The few other *pufM* sequences recovered in this study were closely related to the alphaproteobacterial genera *Roseobacter, Jannaschia, Roseovarius, Sulfitobacter, Loktanella* and to *Luminiphilus* in the *Gammaproteobacteria* (Supplementary Figure S3). These bacteria correspond to aerobic anoxygenic phototrophs (AAP) which are known from previous studies to represent marine, halophilic and salt-dependent bacteria in the top oxic layer of sandy or muddy intertidal sediments (Shiba et al., 1979; Buchan et al., 2005; Ranchou-Peyruse et al., 2006; Spring et al., 2013).

## Discussion

In this study, we used a combination of chemical, molecular and NanoSIMS analyses to investigate natural purple sulfur bacterial mats from the intertidal sediments of the Roscoff Aber Bay and estimate carbon and nitrogen fluxes at the submicrometric scale. We discovered that purple bacteria belonging to the *Thiohalocapsa* cluster represented almost the entire fraction of the expressed *pufM* genes. In addition, the coupled NanoSIMS/CSIA-FAME approach led us to conclude that members of the genus *Thiohalocapsa* were the most photosynthetically active bacteria in the studied mats. As pointed out recently by Gao et al. (2016), NanoSIMS is a handy tool to investigate the functions and process of microorganisms especially in complex natural environments but yet requires further development. We believe that validation of NanoSIMS fluxes by CSIA is a very promising combination in revealing substrate uptake at the single cell level.

### Bacterial Community Composition of Purple Sulfur Mats

The phylogenetic analysis showed that purple bacterial mats were dominated by very few species. Based on 16S rRNA gene library, the most abundant clone was closely related to a *Pseudomonas* strain. Whilst this finding is not completely within the scope of the present study, it is interesting to note that this dominant sequence was found in several different samples, suggesting that the corresponding organism is favored by the modified chemical and physical gradients occurring at the sediment surface and/or takes advantage of the development of purple bacterial mats. Other dominant 16S rRNA clones were mainly affiliated with the family *Chromatiaceae* and most closely related to *Thiohalocapsa* and *Marichromatium*. Members of the genera *Marichromatium* and *Thiohalocapsa* are typical purple sulfur bacteria isolated from or detected in shallow coastal habitats such as the Roscoff Aber Bay where they are exposed to diel cycles of light intensity and temperature (Bolhuis and Stal, 2011; Caumette et al., 1991; Tank et al., 2011). Overall, *pufM* sequences that we obtained mainly grouped within the *Thiohalocapsa* clade. Several lines of evidence indicate that *Thiohalocapsa*-related bacteria were the main purple sulfur bacteria in the mats. First, Tank et al. (2009) demonstrated an overall high consistency of tree topologies of *pufM* and 16S rRNA gene, allowing a phylogenetic classification of purple sulfur bacteria to the genus and in many cases to the species level. Second, *Thiohalocapsa*-related sequences were prominent in the DNA- and rRNA based *pufM* libraries. And third, microcolonies viewed on NanoSIMS images were mainly composed by spherical cells typical of the *Thiohalocapsa* species described to date (Caumette et al., 1991; Imhoff et al., 1998; Anil Kumar et al., 2009). Minor phylotypes of purple sulfur bacteria and aerobic anoxygenic phototrophic bacteria were detected in the mat samples. Nucleotide sequence similarities of these phylotypes showed different degrees of relatedness (from 84 to 96% similarity) to recognized species. The majority of them could be assigned to uncultured *Chromatiaceae* and to aerobic anoxygenic phototrophic alpha- and *Gammaproteobacteria* AAPs related to the genera *Roseobacter clade* and *Luminiphilus*, respectively (Labrenz et al., 1999; Wagner-Dobler et al., 2003; Van Trappen et al., 2004; Macian et al., 2005; Spring et al., 2013). The coexistence of aerobic and anaerobic anoxygenic phototrophs has been reported in bacterial mats covering marine and saline sediments (Ranchou-Peyruse et al., 2006; Thiel et al., 2010). AAP bacteria have been shown to be abundant and active in marine oxygenated waters (Béjà et al., 2002; Koh et al., 2011; Boeuf et al., 2013) and it is not clear whether they are active in oxygen-limited or anoxic conditions. In this study, only one *pufM* sequence from AAP bacteria (OTU10) was recovered in the cDNA-based libraries, suggesting that most of detected AAP bacteria were not able to grow photosynthetically under the low oxygen levels occurring at the mat surface.

### Structure and Heterogeneity of the *Thiohalocapsa* Mats

NanoSIMS images allowed the detection of 1024 ROIs based on the typical coccoïd shape of *Thiohalocapsa*-like cells. The most remarkable feature of the microbial mats was the relative homogeneity of morphotypes characterized by the presence of sulfur globules inside the cells (**Figure 4A**). They result from the transient storage of sulfur during oxidation of sulfide (Caumette et al., 1991). As the likely result of the sample preparation procedure for NanoSIMS (i.e., high vacuum, ionization), the sulfur globules were visible as “holes” in the NanoSIMS images. This situation has already been observed in electron micrographs (Frigaard and Dahl, 2009). In that context, the naturally abundant ^14^N (^12^C^14^N-) images were very useful to detect *Thiohalocapsa*-like cells and define ROIs but, surprisingly, it was not possible to detect elemental sulfur using the ^32^S-detector neither within the globules (as sulfur was probably lost during sample preparation) nor in the rest of the cells.

Our data demonstrated that ^13^C enrichments can vary greatly between individual cells from the same species in the same bacterial mat although they are physically close to each other and even within cell clumps. This finding was surprising given the relative homogeneity of the microbial mats in terms of structure, fatty acid composition and bacterial community composition. This has also been observed in natural populations of other phototrophic microorganisms (Musat et al., 2008; Ploug et al., 2010). Musat et al. (2008) reported this situation in anaerobic phototrophic bacteria and hypothesized that the observed differences could be explained by the presence of physiologically distinct populations of phylogenetically identical or closely related organisms or by differences in gene expression among individual cells. In this study, ^13^C and ^15^N uptake rates were positively related which may indicate that the observed heterogeneity between individuals is likely explained by the presence of active and inactive cells, reflecting distinct physiological states or life “traits” (Musat et al., 2008).

### C Assimilation in Natural Purple Sulfur Mats

NanoSIMS analyses showed that the highest δ^13^C were obtained in the light in the presence of pyruvate, suggesting that pyruvate was the preferred photoassimilated carbon source for the *Thiohalocapsa*-dominated mats in our experimental conditions. However, direct comparison of the δ^13^C and δ^15^N values between treatments must be taken cautiously in the light of the final atomic ratio of each substrate used during the incubation. Indeed, the natural concentrations of acetate, pyruvate, bicarbonate and ammonium as well as their natural δ^13^C and δ^15^N must be known in order to properly estimate C & N fluxes. In our experiments, we used initial bicarbonate (1.3 mM; final isotopic ratio of 33.5 atom%) and ammonium levels (30.8 μM; final isotopic ratio of 86.9 atom%) corresponding to mean values measured in water covering biofilms and that are typical mean values recorded in August (Hubas et al., 2006a). Since we were unable to measure the initial concentrations of acetate and pyruvate before the incubation experiments, these values were taken from the literature. Pyruvate is an important intermediate for microbial metabolism but it is involved exclusively in intracellular reactions (Sansone, 1986). As a consequence, its natural concentrations in pore waters is generally very low (Sansone, 1986; Sawyer and King, 1993; Heuer et al., 2006) thought it can be locally abundant if cell lysis is important or adsorbed onto extracellular polymeric substances through ketal links (Sutherland, 2005). Acetate is generally more abundant than pyruvate in sediments. While most studies agree that acetate concentrations are about 50 μM in the oxic zone of the sediment, they may increase significantly (up to 2 mM) when the environment becomes anoxic (Albert and Martens, 1997). We therefore used concentration estimates for pyruvate (70 μM) and acetate (1000 μM) which correspond to final isotopic ratios of 33.5 and 86.9 atom%, respectively. Using these final atomic ratios, our data showed that *Thiohalocapsa*-related bacteria preferentially assimilate acetate and bicarbonate in the light. Their uptakes of acetate, bicarbonate and pyruvate under light conditions are in agreement with the substrate utilization of *Thiohalocapsa* species in culture under anaerobic conditions when sulfide is present (Caumette et al., 1991; Imhoff et al., 1998; Anil Kumar et al., 2009), suggesting that anoxic conditions prevailed in the mats during the incubation experiments. To our knowledge, no studies have compared the assimilation rates of carbon sources by *Thiohalocapsa* species either in culture or in the natural environment. Here, additionally to their potential nutritional preferences under light, we also showed that acetate assimilation, although slower, was still efficient in the dark while uptakes of bicarbonate and pyruvate were minor in the same conditions. Chemoorganotrophic growth in micro-oxic conditions in the dark has been reported in *T. halophila* with pyruvate (Caumette et al., 1991) and in other *Chromatiaceae* with acetate and/or pyruvate (Imhoff, 2014). This suggests that micro-aerobic conditions also occur in *Thiohalocapsa*-dominated mats. The coexistence of micro-aerobic and anoxic zones at the micrometer scale in the investigated biofilms may also have contributed to the observed heterogeneity in the uptake of labelled substrates as revealed by NanoSIMS analysis. The ability of *Thiohalocapsa*-related bacteria to use acetate, one of the most important intermediates of anaerobic degradation of organic matter, during diurnal cycles of the light regime in oxic to anoxic conditions may likely explain their bloom formation in coastal microbial mats of Roscoff Aber Bay.

Using our single cell NanoSIMS fluxes (**Figure 6**), we extrapolated the bicarbonate fixation rates (mean CO3L fluxes – mean CO3D fluxes) to the whole community assuming a mean cell density of 7.5 × 1011 cells.g sediment^-1^ as evaluated using BChl *a* levels (Hubas et al., 2013), a BChl *a* content of 0.01991 mg Bchl *a*.mg C^-1^ (Overmann, 1997), and a cell carbon content of 20 fgC.cell^-1^ (Lee and Fuhrman, 1987). The result (1.95 ± 0.14 10^-7^ gC.g sediment^-1^.h^-1^) is close to the gross CO_2_ fixation rate (2.7 ± 0.1 10^-7^ gC.g sediment^-1^.h^-1^) of *Thiohalocapsa*-dominated mats measured using benthic chambers in a previous study (Hubas et al., 2013). In this calculation, we considered a mean carbon flux of 27.3 ± 0.9 mgC m^-2^ h^-1^, a chamber surface of 0.71 m^2^, a volume of 1 m^2^ of biofilm of 1000 cm^3^ (width and length = 100 cm, depth = 0.1cm) and a sediment density of 1 g.cm^-3^). Both these estimations are also in line with those (0.18 10^-7^ gC.g sediment^-1^.h^-1^) of purple sulfur bacterial mats from the Orkney islands (Wieland et al., 2003) assuming a O:C ratio of 1 during respiration and the same g to m^2^ conversion factor as above. This confirms that single-cell fixation rates may be useful to estimate carbon fluxes at the biofilm scale.

## Conclusion

The combination of analytical chemistry as well as molecular ecology methods together with NanoSIMS imaging allowed the quantification of carbon and nitrogen fluxes at the submicrometric scale. Here, we demonstrated that *Thiohalocapsa*-dominated mats in the Roscoff Aber Bay assimilated a substantial amount of dissolved bicarbonate although they probably favor a photoheterotrophic lifestyle for their growth. In the future, more precise quantitative C and N budgets for purple sulfur bacterial mats could be constructed. Nevertheless, taking into account their wide dispersion in intertidal sediments, as recently evidenced by spectral reflectance (Hubas et al., 2011), this study gives the way to the hitherto unsuspected importance of purple sulfur bacteria in the coastal carbon biogeochemical fluxes of coastal environments.

## Author Contributions

Project PI: CH; field work: CH, CJ, and BJ; laboratory measurements: DB (molecular), NT (fatty acids), YB (chemistry); NanoSIMS measurements and analysis: CH and CJ; statistical analysis: CH and DB (molecular); manuscript writing: CH and CJ; manuscript revision: CH, CJ, and BJ.

## Conflict of Interest Statement

The authors declare that the research was conducted in the absence of any commercial or financial relationships that could be construed as a potential conflict of interest.
