# Supplementary material for: A Nanoscale Study of Carbon and Nitrogen Fluxes in Mats of Purple Sulfur Bacteria: Implications for Carbon Cycling at the Surface of Coastal Sediments
Source: Front Microbiol. 2017 Oct 24;8:1995. doi: 10.3389/fmicb.2017.01995 (PMC5660696; doi:10.3389/fmicb.2017.01995)
Supplement: Supplementary file 1 [file Presentation_1.pdf]

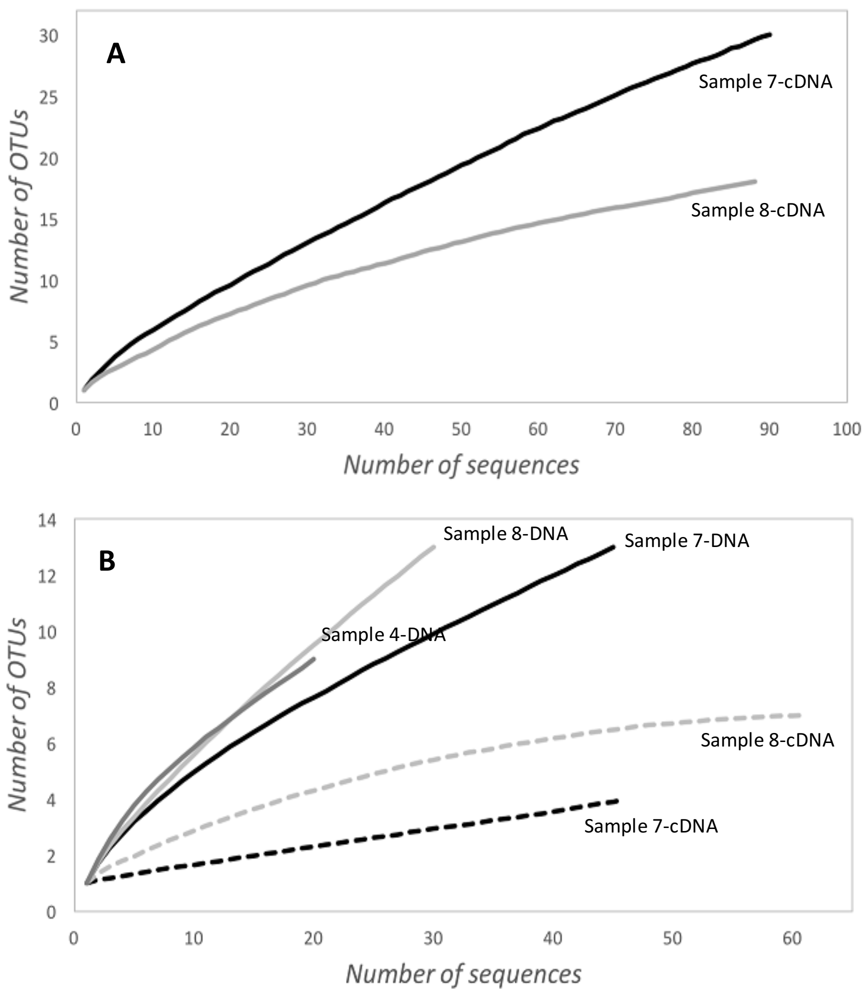

Suppl. Fig. 1. Rarefaction curves of the bacterial 16S rRNA gene (A) and pufM (B) diversity in Roscoff Aber Bay mat samples. Note that cDNA-based clones were generated for both genes while DNA-based clones were additionally obtained for the pufM gene (see Suppl. Tables 1 and 2). OTUs were separated at 97% and 94% identity for 16S rRNA gene and pufM diversity, respectively.

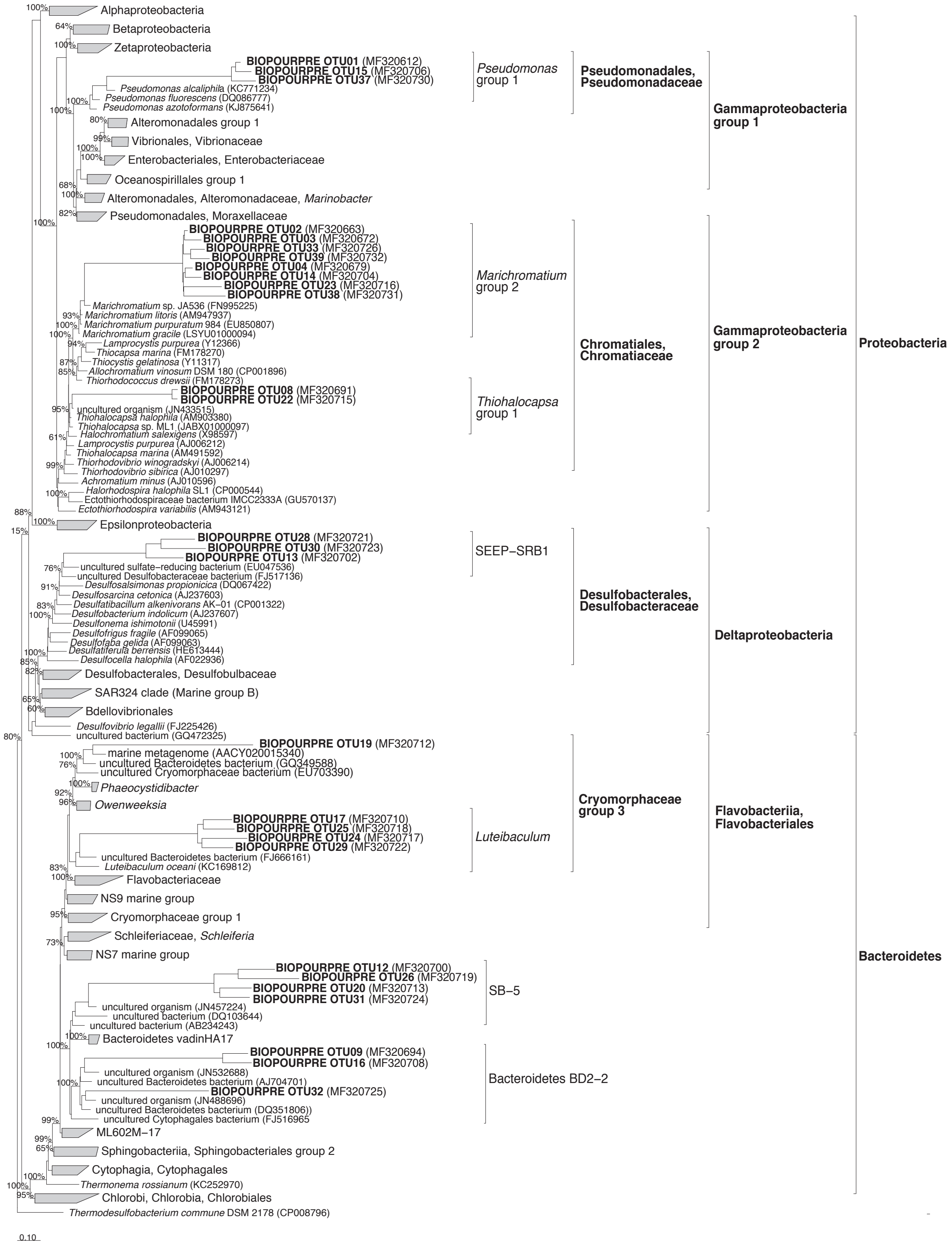

Suppl. Fig. 2. Phylogenetic tree showing the relationships between bacterial 16S rRNA gene sequences retrieved from the Roscoff Aber Bay mats (in bold) and relatives retrieved in the SILVA database. The tree is based on the reference SILVA tree to which sequences obtained in this study (mean length, 935 bp) were added by ARB_PARSIMONY. Non informative taxa were removed afterward for clarity purposes. The scale bar represents dissimilarity between nucleotide positions.

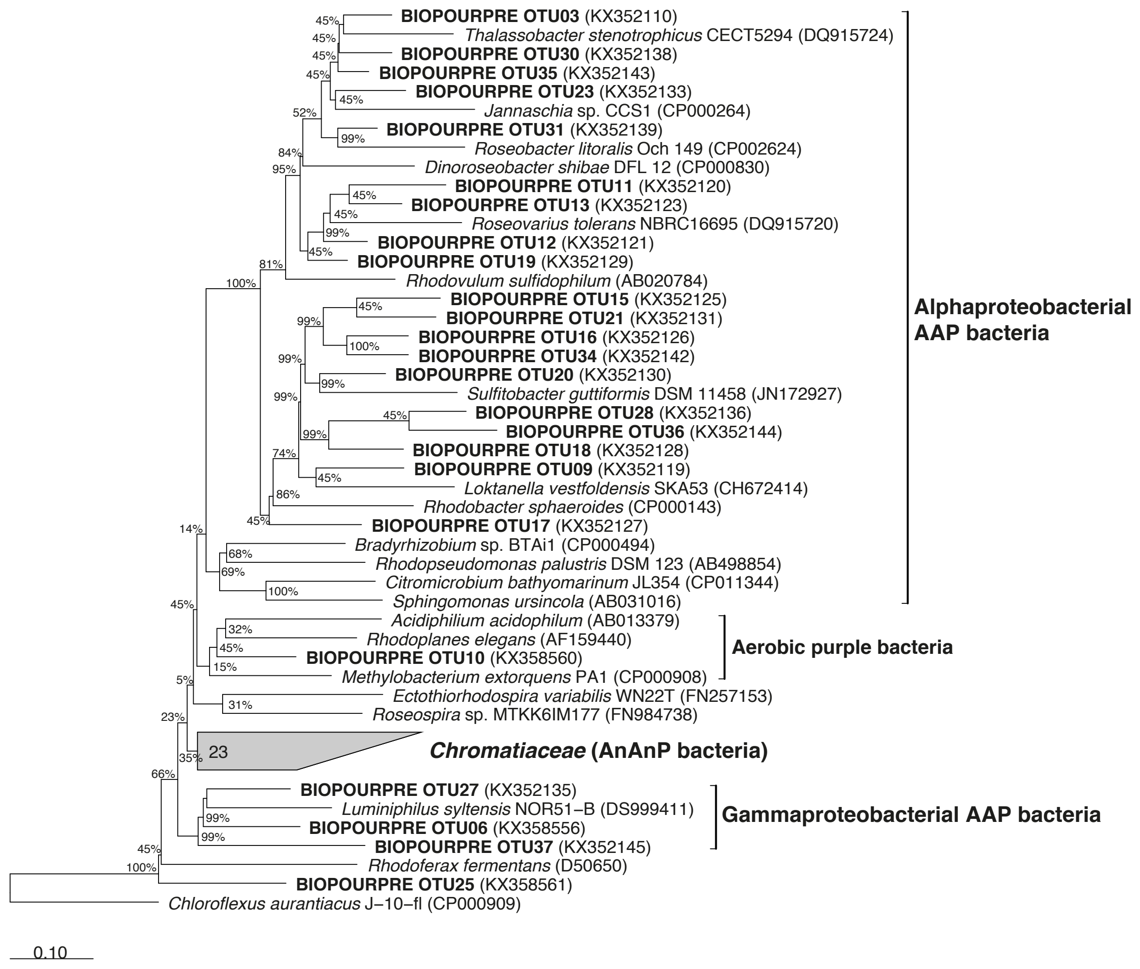

Suppl. Fig. 3. Phylogenetic tree showing the relationships between *pufM* gene sequences of aerobic anoxygenic phototrophic bacteria retrieved from the Roscoff Aber Bay mats (in bold) and relatives retrieved in GenBank. The tree is based on a Bayesian tree to which sequences obtained in this study (185 bp) were added by ARB_PARSIMONY. The scale bar represents dissimilarity between nucleotide positions.
