# Supplementary material for: A Nanoscale Study of Carbon and Nitrogen Fluxes in Mats of Purple Sulfur Bacteria: Implications for Carbon Cycling at the Surface of Coastal Sediments
Source: Front Microbiol. 2017 Oct 24;8:1995. doi: 10.3389/fmicb.2017.01995 (PMC5660696; doi:10.3389/fmicb.2017.01995)
Supplement: Supplementary file 2 [file Table_1.pdf]

Supplementary Table 1. Operational taxonomic unit (OTU) table of the *pufM* clones obtained in the DNA- and cDNA-based libraries from Roscoff Aber Bay mat samples s4, S7 and s8.

OTU	Accession number	Number of clones	Relative abundance in the DNA-based libraries (%)				Relative abundance in the cDNA-based libraries (%)			BlastN identification	Mean identity (%)
			Total	s4	s7	s8	Total	s7	s8		
OTU01	KX352089	129	39	0	49	50	85	94	79	Uncultured *Thiohalocapsa* sp. clone S1_OTU_8_RP	89
OTU02	KX352101	15	14	30	9	10	2	0	3	Uncultured *Thiohalocapsa* sp. clone S1_OTU_8_RP	94
OTU03	KX352110	8	8	0	18	0	0	0	0	Uncultured bacterium clone Berpuf64	95
OTU04	KX352115	5	5	25	0	0	0	0	0	Uncultured *Thiohalocapsa* sp. clone S1_OTU_8_RP	91
OTU05	KX358555	5	0	0	0	0	5	2	7	Uncultured bacterium clone S4_28	89
OTU06	KX358556	3	0	0	0	0	3	2	3	Uncultured bacterium clone T0_OTU_21_RP	85
OTU07	KX352117	3	3	15	0	0	0	0	0	Uncultured *Thiohalocapsa* sp. clone S1_OTU_8_RP	89
OTU08	KX358559	2	0	0	0	0	2	0	3	Uncultured *Thiohalocapsa* sp. clone S1_OTU_8_RP	91
OTU09	KX352119	2	2	0	0	7	0	0	0	Uncultured bacterium clone SIPP3-1	98
OTU10	KX358560	2	0	0	0	0	2	0	3	Uncultured bacterium clone T11_OTU_9_RP	89
OTU11	KX352120	2	2	0	4	0	0	0	0	Uncultured bacterium clone 14-6	86
OTU12	KX352121	2	2	0	2	3	0	0	0	Uncultured bacterium clone Berpuf16	92
OTU13	KX352123	1	1	0	2	0	0	0	0	*Roseovarius tolerans* strain NBRC16695	92
OTU14	KX352124	1	1	0	0	3	0	0	0	Uncultured *Thiohalocapsa* sp. clone S1_OTU_8_RP	89
OTU15	KX352125	1	1	0	2	0	0	0	0	Alphaproteobacterium R2A130	89
OTU16	KX352126	1	1	0	0	3	0	0	0	Uncultured bacterium clone A5PM-75	90
OTU17	KX352127	1	1	0	2	0	0	0	0	Uncultured alphaproteobacterium clone JAP15BAP	89
OTU18	KX352128	1	1	0	0	3	0	0	0	Uncultured *Rhodobacteraceae* clone T0_OTU_22_RP	89
OTU19	KX352129	1	1	0	0	3	0	0	0	Uncultured bacterium clone Berpuf16	92
OTU20	KX352130	1	1	0	0	3	0	0	0	Uncultured bacterium clone A5PM-99	92
OTU21	KX352131	1	1	0	2	0	0	0	0	Uncultured bacterium	89
OTU22	KX352132	1	1	0	0	3	0	0	0	Uncultured bacterium clone Berpuf18	87
OTU23	KX352133	1	1	0	0	3	0	0	0	Uncultured bacterium clone A5PM-297	90
OTU24	KX352134	1	1	0	0	3	0	0	0	*Lamprocystis purpurea*	91
OTU25	KX358561	1	0	0	0	0	1	2	0	*Thiohalocapsa marina* type strain JA142T	89
OTU26	KX358562	1	0	0	0	0	1	0	2	Uncultured *Thiohalocapsa* sp. clone S1_OTU_8_RP	91
OTU27	KX352135	1	1	5	0	0	0	0	0	Uncultured bacterium clone B05	85
OTU28	KX352136	1	1	5	0	0	0	0	0	Uncultured bacterium clone NS_WP46	91
OTU29	KX352137	1	1	0	2	0	0	0	0	Uncultured marine bacterium clone C33	84
OTU30	KX352138	1	1	0	0	3	0	0	0	Alphaproteobacterium MBIC3951	92
OTU31	KX352139	1	1	0	2	0	0	0	0	*Roseobacter litoralis* Och 149	96
OTU32	KX352140	1	1	5	0	0	0	0	0	Uncultured *Thiohalocapsa* sp. clone S1_OTU_8_RP	94
OTU33	KX352141	1	1	5	0	0	0	0	0	Uncultured *Thiohalocapsa* sp. clone S1_OTU_8_RP	88
OTU34	KX352142	1	1	0	2	0	0	0	0	Uncultured bacterium clone st.1-64n	87
OTU35	KX352143	1	1	5	0	0	0	0	0	Uncultured bacterium clone Berpuf18	92
OTU36	KX352144	1	1	5	0	0	0	0	0	Uncultured bacterium clone QL-0-SP2	92
OTU37	KX352145	1	1	0	2	0	0	0	0	Uncultured proteobacterium clone T0_OTU_19_RP	92
